# Proteomic and metabolomic characterization of CHO DP-12 cell lines with different high passage histories

**DOI:** 10.1186/1753-6561-5-S8-P92

**Published:** 2011-11-22

**Authors:** Tim Beckmann, Tobias Thüte, Christoph Heinrich, Heino Büntemeyer, Thomas Noll

**Affiliations:** 1 Institute of Cell Culture Technology, Bielefeld University, 33615 Bielefeld, Germany

## Background

For industrial pharmaceutical protein production fast growing, high producing and robust cell lines are required. To select more pH shift permissive and fast growing sub-populations, the CHO DP-12 (ATCC clone #1934) cell line, an anti-IL8 antibody producing CHO K1 (DHFR^-^) clone, was continuously subcultured at high viability (>90 %) for more than four hundred days in shaking flasks using a chemically defined medium. During this long-term cultivation there was a repeated shift in pH and most robust and fast growing cells became accumulated [1]. Cell samples were cryopreserved at four different time points, after 21, 95, 165 and 420 days (in the following named sub-populations (SP)). The effects of long-term passaging before cryopreservation correlating with an increase in specific growth rate as well as changes in product formation and metabolism were examined in parallel bench-top bioreactor cultivation of SP21, SP95, SP165 and SP420 sub-populations. During exponential growth phase samples were taken for the analyses of differences in intracellular metabolites and protein expression (Please consider article “Growth characterization of CHO DP-12 cell lines with different high-passage histories” by Heinrich *et al.* in this issue for a detailed discussion of long-term cultivation, changes in specific growth rate, product formation and metabolic shifts).

## Material and methods

The CHO DP-12 cell line was cultivated in chemically defined, animal component-free medium TC 42 (TeutoCell AG) with addition of 8 mM glutamine and 200 nM methotrexate. For first steps of suspension adaption PowerCHO-2 (Lonza AG) medium was used. Parallel bioreactor cultivations were performed in 2 L bench-top vessels with an initial working volume of 1.2 L. Cultivation parameters were closed-loop controlled and set to 37 °C, pH 7.1 and 40 % DO of air saturation. Cell counting and determination of viability was performed using a CEDEX system (Innovatis-Roche AG). Samples for metabolome and proteome analysis were taken from parallel bioreactor cultivations of the four sub-populations during exponential growth phase. Changes in the protein expression were analyzed by differential two-dimensional gel electrophoresis (GE Healthcare) with four technical replicates inclusive dye swap, respectively. Image processing and statistical evaluation was carried out with Delta 2D 4.2 software (Decodon GmbH). All differentially expressed protein spots were successfully identified using an ultrafleXtreme MALDI-TOF-TOF (Bruker Daltonics). For the analysis of intracellular metabolites an in-house developed fast-filtration quenching procedure was used to generate samples for GC-MS and LC-MS measurements [2]. For GC-MS analyses keto- and aldehyde functions were converted into their oxime derivatives using methoxyamine hydrochloride. Other relevant functions like amines, carboxylic acids or hydroxyls were masked with trimethylsilyl groups resulting in volatile derivatives. The nucleotide concentrations were determined by a HILIC-MS method using a MonoChrom diol column (Varian).

## Results

Based on the expression of 1377 detected proteins spots the four sub-populations could be clearly separated from each other in a principle component analysis. An ANOVA analysis (α ≤ 0.05, false significant proportion ≤ 0.01) revealed 43 protein spots with significantly different abundance. Hierarchical clustering and examination of fold-changes of significantly different expressed proteins showed that the quantity of different proteins and the degree of change increased with the number of passages before cryopreservation. Between SP21 and SP95 only three protein spots were detected with an at least two-fold change. For SP196 and SP420 seven and 41 spots showed a ratio with an absolute value of two in comparison with SP21, respectively. In a follow up analysis the correlation of protein expression and the number of passages was examined. A template matching approach (R ≥ 0.98) [3] revealed 23 proteins whose abundance was linearly correlated with the number of days before cryopreserving the cells. Among these spots anti-stress proteins, candidates involved in protein folding, glycolytic enzymes and also proteins participating in transcription regulation, mRNA processing, cytoskeleton formation, protein biosynthesis as well as folate and purine metabolism were identified. In addition, there was a set of 10 unique proteins which showed an inverted correlation with the number of passages. The results from proteomic analysis indicate that the four subpopulations not only differ in protein abundances directly related to cell growth, but also show differences in diverse aspects of cellular protein expression.

The analysis of intracellular metabolites revealed a positive correlation between the uptake rates of extracellular metabolites and their intracellular pool size. Interestingly, this trend is not fulfilled for a couple of the investigated metabolites: For example, the aspartate pool increases while the uptake of extracellular aspartate decreases although it is not at limiting concentrations. This indicates that the replenishment by intracellular pathways is favored over the uptake of extracellular substrate in this case. Furthermore, an increase of the adenine as well as guanine energy charge was observed for an increasing number of passages. Both, adenosine- and guanosine-5'-triphosphate, are necessary for anabolic reactions as well as protein synthesis. They may also play a role in apoptosis by inhibiting the formation of the apoptosome [4] and therefore might be one factor for the observed increase in specific growth rate.

By merging the proteomic findings with the measurements of intracellular metabolite pools we gained a better understanding of factors which let a production cell line grow faster and be more robust against pH shifts. This example shows that the analysis of protein expression combined with measurements of intracellular pool sizes may give additional hints for cell line, process and media development in addition to classical approaches.
